# Stability of the Virome in Lab- and Field-Collected Aedes albopictus Mosquitoes across Different Developmental Stages and Possible Core Viruses in the Publicly Available Virome Data of *Aedes* Mosquitoes

**DOI:** 10.1128/mSystems.00640-20

**Published:** 2020-09-29

**Authors:** Chenyan Shi, Lu Zhao, Evans Atoni, Weifeng Zeng, Xiaomin Hu, Jelle Matthijnssens, Zhiming Yuan, Han Xia

**Affiliations:** a Center Lab of Longhua Branch and Department of Infectious Disease, The Second Clinical Medical College, Jinan University (Shenzhen People's Hospital), Shenzhen, China; b Post-doctoral Scientific Research Station of Basic Medicine, Jinan University, Guangzhou, China; c Department of Microbiology, Immunology and Transplantation, Laboratory of Clinical and Epidemiological Virology, Laboratory of Viral Metagenomics, Rega Institute, KU Leuven, Leuven, Belgium; d Key Laboratory of Special Pathogens and Biosafety, Wuhan Institute of Virology, Chinese Academy of Sciences, Wuhan, Hubei, China; e University of Chinese Academy of Sciences, Beijing, China; f Liwan Center for Disease Control and Prevention, Guangzhou, Guangdong, China; g College of Life Science, South-Central University for Nationalities, Wuhan, China; University of Delhi

**Keywords:** *Aedes* mosquito, Guadeloupe mosquito virus, Phasi Charoen-like phasivirus, core virome, environment-derived core virome, vertically transmitted core virome

## Abstract

*Aedes* mosquitoes can efficiently transmit many pathogenic arboviruses, placing a great burden on public health worldwide. In addition, they also carry a number of insect-specific viruses (ISVs), and it was recently suggested that some of these ISVs might form a stable species-specific “core virome” in mosquito populations. However, little is known about such a core virome in laboratory colonies and if it is present across different developmental stages. In this study, we compared the viromes in eggs, larvae, pupae, and adults of Aedes albopictus mosquitoes collected from a lab colony and compared each to the virome of different developmental stages collected in the field. The virome in lab-derived *A. albopictus* was very stable across all stages, consistent with a vertical transmission route of these viruses, and formed a possible “vertically transmitted core virome.” The different stages of field-collected *A. albopictus* mosquitoes also contained this stable vertically transmitted core virome, as well as another set of viruses (e.g., viruses distantly related to Guadeloupe mosquito virus, Hubei virga-like virus 2, and Sarawak virus) shared by mosquitoes across different stages, which might represent an “environment-derived core virome.” To further study this core set of ISVs, we screened 48 publicly available SRA viral metagenomic data sets of mosquitoes belonging to the genus *Aedes*, showing that some of the identified ISVs were identified in the majority of SRAs and providing further evidence supporting the core-virome concept.

**IMPORTANCE** Our study revealed that the virome was very stable across all developmental stages of both lab-derived and field-collected Aedes albopictus. The data representing the core virome in lab *A. albopictus* proved the vertical transmission route of these viruses, forming a “vertically transmitted core virome.” Field mosquitoes also contained this stable vertically transmitted core virome as well as additional viruses, which probably represented “environment-derived core virome” and which therefore were less stable over time and geography. By further screening publicly available SRA viral metagenomic data sets from mosquitoes belonging to the genus *Aedes*, some of the identified core ISVs were shown to be present in the majority of SRAs, such as Phasi Charoen-like phasivirus and Guadeloupe mosquito virus. How these core ISVs influence the biology of the mosquito host and arbovirus infection and evolution deserves to be further explored.

## Abstract (precis)

Our study revealed that the virome was very stable across all developmental stages of both lab-derived and field-collected Aedes albopictus. The data representing the core virome in lab *A. albopictus* proved the vertical transmission route of these viruses, forming a “vertically transmitted core virome.” Field mosquitoes also contained this stable vertically transmitted core virome as well as additional viruses, which probably represented “environment-derived core virome” and which therefore were less stable over time and geography. By further screening publicly available SRA viral metagenomic data sets from mosquitoes belonging to the genus *Aedes*, some of the identified core ISVs were shown to be present in the majority of SRAs, such as Phasi Charoen-like phasivirus and Guadeloupe mosquito virus. How these core ISVs influence the biology of the mosquito host and arbovirus infection and evolution deserves to be further explored.

## INTRODUCTION

Mosquitoes are highly diverse and widely disseminated. They occupy numerous biotopes and are potential vectors for several pathogenic arboviruses. Specifically, *Aedes* spp. pose a great threat to global public health. This is due to their traits of ecological plasticity that include egg diapause (1), versatility in using natural and/or urban breeding spots (2), and opportunistic feeding patterns (3, 4), which might have promoted their dispersion and adaptation to new, uncolonized territories that range from tropical to temperate regions (5). Moreover, a large number of pathogenic arboviruses, such as dengue virus (DENV), yellow fever virus (YFV), Zika virus (ZIKV), and chikungunya virus (CHIKV), are efficiently transmitted by *Aedes* mosquitoes, mainly A. aegypti and *A. albopictus* (6). In particular, DENV epidemics are a major public health concern in Guangdong Province of southern China, especially in its capital city, Guangzhou (GZ). The dengue fever cases in Guangzhou represented 50% of the national incidence between 1978 and 2011 (7), and there was an explosive outbreak in 2014 with 45,224 reported dengue fever cases (8). *A. albopictus* is among the most invasive mosquitoes and is widely distributed in China. It is the main vector for DENV in China and the sole vector in Guangzhou (9, 10).

In light of the holobiont concept developed in recent years, mosquito-associated microbial communities play an important role in host biology, which may provide new strategies for mosquito and arbovirus control (11). Mosquitoes are holometabolous insects whose life cycle goes through four separate stages, including egg, larva, and pupa stages in an aquatic habitat and a subaerial adult stage. A continuum of bacteria from the aquatic environment to immature stages and adult mosquitoes has been suggested (12), but bacterial clearance occurs during mosquito metamorphosis from pupae to adults (13). In addition, the nutrients produced by symbiotic bacteria are very important for larval development (14) and larval bacteria can influence immune responses and vector competence in adult mosquitoes (15).

However, little is known about the virome community dynamics in mosquitoes during holometabolous development. Some studies have performed viral metagenomics on field mosquitoes collected from different countries (including the United States, Puerto Rico, China, Kenya, Australia, Sweden, etc.), mainly focusing on novel virus discovery (16–24). Some newly discovered mosquito-specific viruses (MSVs) have been proposed as future biological control agents against arboviruses (25–27) or as novel vaccine platforms (27) or used in diagnostic assays in chimeric virus formation with structural protein genes from arboviruses (27). In a recent study, we showed the presence of a relatively stable “core eukaryotic virome” in adult field A. aegypti and Culex quinquefasciatus mosquitoes collected in Guadeloupe (28). However, the presence of insect-specific viruses (ISVs) in mosquito lab colonies is poorly studied, which limits our understanding of their role in the development and physiology of mosquitoes. On the other hand, knowledge of the normal healthy background virome as a reference will allow us to distinguish between inherent vertically transmitted components and components acquired from the environment of field mosquitoes, which will enable the identification of potential viral pathogens and improve the reliability of risk assessment using lab mosquitoes.

The first aim of this study was to analyze the virome structure of *A. albopictus* mosquitoes during distinct developmental stages in a laboratory-derived colony originally obtained in Jiangsu Province, China. Second, we compared these lab colony-derived viromes with the viromes of field-collected *A. albopictus* mosquitoes from Guangzhou (Guangdong Province, China), representing locations that are approximately 1,300 kilometers apart. Third, we conducted a comparative meta-analysis using comparisons between the viruses identified in this study and 48 publicly available virome SRA data sets of *Aedes* mosquitoes. Finally, phylogenetic analyses were performed on the following three selected viruses: *Aedes* phasmavirus (APV), which was highly present in both field and lab *A. albopictus* from China; Guadeloupe mosquito virus (GMV)-related viruses; and Phasi Charoen-like phasivirus (PCLPV), which was found in the majority of investigated virome data sets.

## RESULTS

### Comparison of the eukaryotic viromes in lab- and field-collected *A. albopictus*.

**FIG 1:**
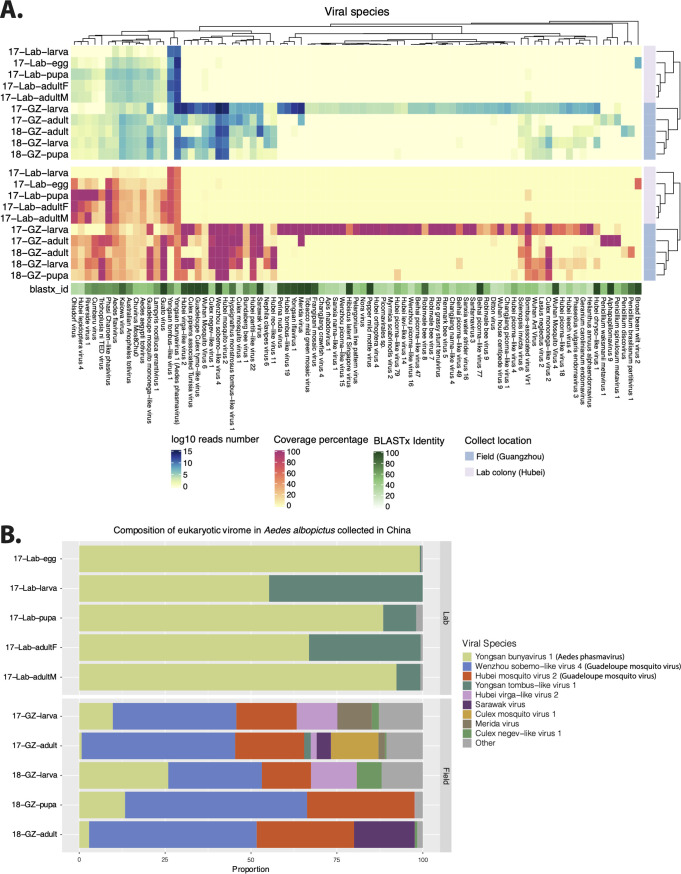
Eukaryotic viral genomes in wild and lab-derived Aedes albopictus pools. (A) The heat maps show the total number of mapped reads on a log_10_ scale (upper panel) and length coverage (lower panel) of assembled contigs of each virus. The hierarchical clustering is based on the Bray-Curtis distance matrix calculated from the number of log_10_ reads. The viral species names shown in the heat map are from the taxonomic annotation by DIAMOND and KronaTools. For each of the contigs assigned to a particular species, the open reading frame (ORF) with the highest BLASTx identity to a reference sequence was taken, and the median identity of these different ORFs is shown in the shaded green bar. (B) Relative abundances of the viral species in each pool based on the number of reads.

Pools of eggs, larvae, pupae, and (male/female) adults from a lab colony in Wuhan, as well as pools of larvae, pupae, and adults from the field in Guangzhou underwent metagenomic sequencing. Averages of 26.2 million trimmed reads were obtained per pool (Table 1) and were *de novo* assembled into 1,657,229 contigs in total. The clustering of 71,303 contigs longer than 500 bp from all samples at 95% nucleotide (nt) identity over 80% of the read length resulted in 56,419 representative contigs. According to BLASTx annotation results obtained using DIAMOND, the majority (93%) of the representative contigs belonged to Eukaryota (mainly derived from the mosquito host genome) and 179 contigs were assigned as eukaryotic viruses. Eukaryotic viral reads in each pool occupied 0.2% to 1.8% of the trimmed reads, except for the 17-GZ-larva pool, which contained 15.8% viral reads. Most of the eukaryotic viral contigs belonged to 80 different viruses (including several viruses with segmented genomes), although some of these had very low similarities to known viruses in the database (Fig. 1A). No known pathogenic arboviruses were identified, and the closest relatives of the identified viruses were generally found in insects or plants. Only 20 of the 80 viral species belonged to an established viral genus or family (e.g., *Flavivirus*, *Iflavirus*, *Phasivirus*, *Quaranjavirus*, *Rhabdoviridae*, *Virgaviridae*, *Totiviridae*, *Nodaviridae*), whereas the remaining viral genomes were most closely related to viruses discovered in recent years that current lack a formal taxonomical classification.

**TABLE 1:** Aedes albopictus from China used for viral metagenomic sequencing

Sample name	Mosquito species	Yr of collection	Habitat	Location	No. of mosquitoes used for sequencing	No. of trimmed reads obtained
17-Lab-egg	*A. albopictus*	2017	Lab	Wuhan, Hubei, China	200	23,801,510
17-Lab-larva	*A. albopictus*	2017	Lab	Wuhan, Hubei, China	250	22,990,930
17-Lab-pupa	*A. albopictus*	2017	Lab	Wuhan, Hubei, China	250	18,066,298
17-Lab-adultF	*A. albopictus*	2017	Lab	Wuhan, Hubei, China	250	20,109,482
17-Lab-adultM	*A. albopictus*	2017	Lab	Wuhan, Hubei, China	250	19,227,638
17-GZ-larva	*A. albopictus*	2017	Field	Liwan district, Guangzhou, China	2,000	29,564,646
17-GZ-adult	*A. albopictus*	2017	Field	Liwan district, Guangzhou, China	1,000	37,766,072
18-GZ-larva	*A. albopictus*	2018	Field	Liwan district, Guangzhou, China	2,000	35,580,984
18-GZ-pupa	*A. albopictus*	2018	Field	Liwan district, Guangzhou, China	500	25,744,126
18-GZ-adult	*A. albopictus*	2018	Field	Liwan district, Guangzhou, China	1,100	29,644,914

The heat map shown in Fig. 1A displays the number of reads mapped against each of the representative (partial) viral genomes as well as the percentage of coverage of each virus per sample (see Table S3 and Fig. S1 in the supplemental material). The virome in field-collected *A. albopictus* from both 2017 and 2018 showed significantly higher richness and diversity than that in lab-derived mosquito pools. The adjusted *P* values of the Wilcoxon test determined for the Chao1 and Shannon indices were 0.012 and 0.008, respectively (Fig. S1). The virome profiles of the different pools of lab colonies were relatively stable, except that several viral species were absent from or were present in low abundance in the larva pool (Fig. 1A). The field-collected larva, pupa, and adult pools collected in GZ in 2018 and the adult pool collected in 2017 also displayed very similar viral communities. However, the larval pool collected in GZ in 2017 contained more than 30 additional unique viral species with almost 100% length coverage that were almost completely absent in the adults collected at the same time and in the same place. Furthermore, the lab and field *A. albopictus* pools had 20 viral species in common. Among them, some contigs distantly related (47% BLASTx identity) to Yongsan bunyavirus 1 (YBV1) were present in all lab- and field-derived mosquito pools with high abundance and length coverage (Fig. 1). Some viruses such as Yongsan tombus-like virus 1 and Phasi Charoen-like phasivirus appeared to be present at higher levels in the lab-derived *A. albopictus* samples. In contrast, reads of several viruses appeared to be present in higher numbers in the field samples, as was the case for Guato virus and Guadeloupe mosquito mononega-like virus (Fig. 1A). Furthermore, field mosquito pools contained many more viruses which were absent in lab-derived samples (Fig. 1A), as exemplified by Wenzhou sobemo-like virus 4 and Hubei mosquito virus 2 (both with close relationship to Guadeloupe mosquito virus) (Fig. 1B).

10.1128/mSystems.00640-20.1 FIG S1 Alpha diversity of viromes in the lab and field Aedes albopictus samples. Download FIG S1, PDF file, 0.2 MB. Copyright © 2020 Shi et al. 2020 Shi et al. This content is distributed under the terms of the Creative Commons Attribution 4.0 International license.

10.1128/mSystems.00640-20.4 TABLE S3 Number of reads of each eukaryotic viral species in each sequencing sample. Download Table S3, XLSX file, 0.01 MB. Copyright © 2020 Shi et al. 2020 Shi et al. This content is distributed under the terms of the Creative Commons Attribution 4.0 International license.

### Prevalence of viruses in public SRA virome data sets derived from *Aedes* mosquitoes.

All 48 available sets of viral metagenomic data representing *Aedes* sp. from the public database (SRA) (see Table S1 in the supplemental material) were further analyzed to determine the conserved prevalence of ISVs in various *Aedes* mosquitoes. These samples were collected from locations on different continents, including the United States, Puerto Rico, Australia, Thailand, Guadeloupe, China, and Kenya. The mosquito species in the majority of the samples was A. aegypti, except for one sample from China (*A. albopictus*) and five from southwestern Australia (*A. alboannulatus* or *A. camptorhynchus*).

10.1128/mSystems.00640-20.2 TABLE S1 SRA datasets used in this study. Download Table S1, XLSX file, 0.01 MB. Copyright © 2020 Shi et al. 2020 Shi et al. This content is distributed under the terms of the Creative Commons Attribution 4.0 International license.

**FIG 2:**
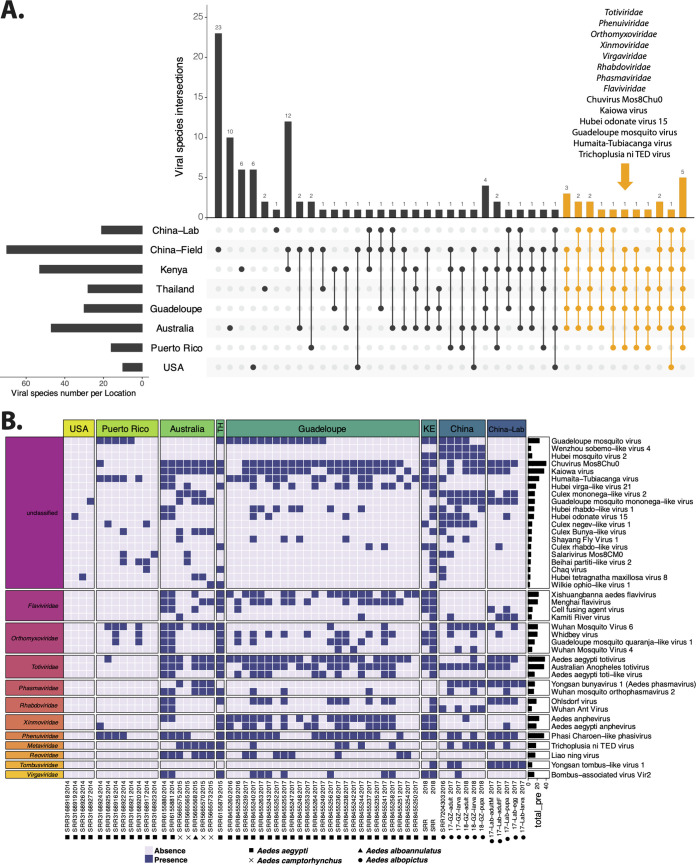
Conservation of viral species in *Aedes* sp. virome data sets. (A) The number of shared viral species among *Aedes* mosquitoes from different locations. (B) The viral species shown in the heat map present in samples from at least three locations and containing at least one contig that is longer than 1,500 bp. The specific viral species was considered to be present in the sample as long as the sample had one contig (>500 bp) assigned to the species.

In order to investigate the potential presence of a “core virome,” we analyzed the presence/absence of each virus across the geographic origins reflected in the available SRA entries. The number of viral species shared among different locations is displayed in Fig. 2A, and virus names corresponding to each overlapping category can be found in Table S5. Twenty viruses belonging to the families *Totiviridae*, *Phenuiviridae*, *Orthomyxoviridae*, *Xinmoviridae*, *Virgaviridae*, *Rhabdoviridae*, *Phasmaviridae*, and *Flaviviridae* and six unclassified viruses (Chuvirus Mos8Chu0, Kaiowa virus, Hubei odonate virus 15, Guadeloupe mosquito virus, Humaita-Tubiacanga virus, Trichoplusia ni TED virus) were identified from at least five locations. Notably, the total number of viral species present in samples from the United States and Puerto Rico was much lower than that in samples from other locations, which could have been due to the presence of different viruses in these samples, less optimal VLP enrichment procedures used, and/or the lower sequencing depths.

10.1128/mSystems.00640-20.5 TABLE S4 Coverage of each eukaryotic viral species in each sequencing sample. Download Table S4, XLSX file, 0.01 MB. Copyright © 2020 Shi et al. 2020 Shi et al. This content is distributed under the terms of the Creative Commons Attribution 4.0 International license.

10.1128/mSystems.00640-20.6 TABLE S5 Presence and absence of viral species in different locations. Download Table S5, CSV file, 0.00 MB. Copyright © 2020 Shi et al. 2020 Shi et al. This content is distributed under the terms of the Creative Commons Attribution 4.0 International license.

The presence or absence of each virus in each of the analyzed virome data sets is shown in the heat map in Fig. 2B. Only the 42 viral species present in a minimum of three locations and containing at least one contig longer than 1.5 kb are displayed in the figure, and they were grouped by collection locations and viral families. *Totiviridae* was the most prevalent viral family, containing two highly abundant species (Aedes aegypti totivirus and Australian Anopheles totivirus) with positivity rates across samples of 63.8% and 60.3%, respectively. Phasi Charoen-like phasivirus belonging to the *Phenuiviridae* was present in all four *Aedes* species and at all locations (except SRAs from the United States, in which only three of the investigated viruses were identified). Among the unclassified viral species, Chuvirus Mos8Chu0 virus and Kaiowa virus were present in 40 and 35 of 58 samples, respectively, and were found in all four *Aedes* species. Guadeloupe mosquito virus was detected in field A. aegypti and *A. albopictus* mosquitoes from Puerto Rico, Thailand, Guadeloupe, Kenya, and China. Humaita-Tubiacanga virus (absent in lab-derived and wild *A. albopictus* from this study) was found in field *A. albopictus* from Yunnan (China) and field A. aegypti from 5 locations.

### Phylogeny of three selected viruses.

Considering the abundance, variance, universality, and number of complete coding genomes of viruses obtained, phylogenetic analysis was conducted on the following three selected viruses: (i) *Aedes* phasmavirus (APV; see below), a novel virus distantly related to YBV1 with high abundance in both field and lab *A. albopictus* samples from China; (ii) Phasi Charoen-like phasivirus (PCLPV), present in all four *Aedes* species and all locations (except the United States) of screened virome data sets; and (iii) Guadeloupe mosquito virus (GMV), occupying the majority of reads in the field-collected *A. albopictus* samples from Guangzhou, widely distributed in SRA data sets and showing a high level of variance. Complete coding viral genomes of APV, PCLPV, and GMV recovered from analyzed virome data sets of *Aedes* mosquitoes as well as corresponding reference genomes from GenBank were used for phylogeny. YBV1 is a newly identified virus reported in 2019 that showed high prevalence in *A. vexans nipponii* from Korea (29) and is classified in the family *Phasmaviridae*. We identified a novel virus, APV, in this family in both lab and field *A. albopictus* samples from China whose closest reference was YBV1. PCLPV, a widely distributed mosquito virus, has been identified from lab colonies, mosquito cell lines, and wild *Aedes* mosquitoes in eight counties or regions (China, Puerto Rico, Thailand, Kenya, the United States, Australia, Guadeloupe, and Brazil) (Table 2). It has been suggested that it is maintained in nature through vertical transmission (30). A recent study has reported that GMV is closely related to Wenzhou sobemo-like virus 4 and Hubei mosquito virus 2 (28). GMV-related viruses were detected only in mosquito samples collected from the field as listed in Table 2 (i.e., were not detected in the mosquito cell lines or lab colonies), indicating that it was likely acquired horizontally from the environment.

**TABLE 2:** Distribution of APV-, PCLPV-, and GMV-related viruses

Source	Region	Origin	APV (related to YBV1)	PCLPV	GMV-related virus
Lab	China	2017-Wuhan-lab	+	+	
	Belgium	2018-Belgium-lab cell line		+	
	United Kingdom	2017-Bristol-lab cell line		+	

Field	China	2016-Zhejiang		+	+
		2017-Guangzhou	+	+	+
		2018-Guangzhou	+	+	+
	Puerto Rico	2014-Puerto Rico		+	+
	Thailand	2008-Thailand		+	
		2015-Thailand		+	+
	Kenya	2018-Kenya1		+	+
		2018-Kenya2		+	+
	United States	2014-United States			
		2015-United States		+	
	Australia	2014-Australia		+	
		2015-Australia	+	+	
		2016-Australia		+	
	Korea	2016-Korea	+		
	Guadeloupe	2016-Guadeloupe		+	+
		2017-Guadeloupe		+	+
	Brazil	2012-Brazil		+	

### Phylogeny of *Aedes* phasmavirus.

**FIG 3:**
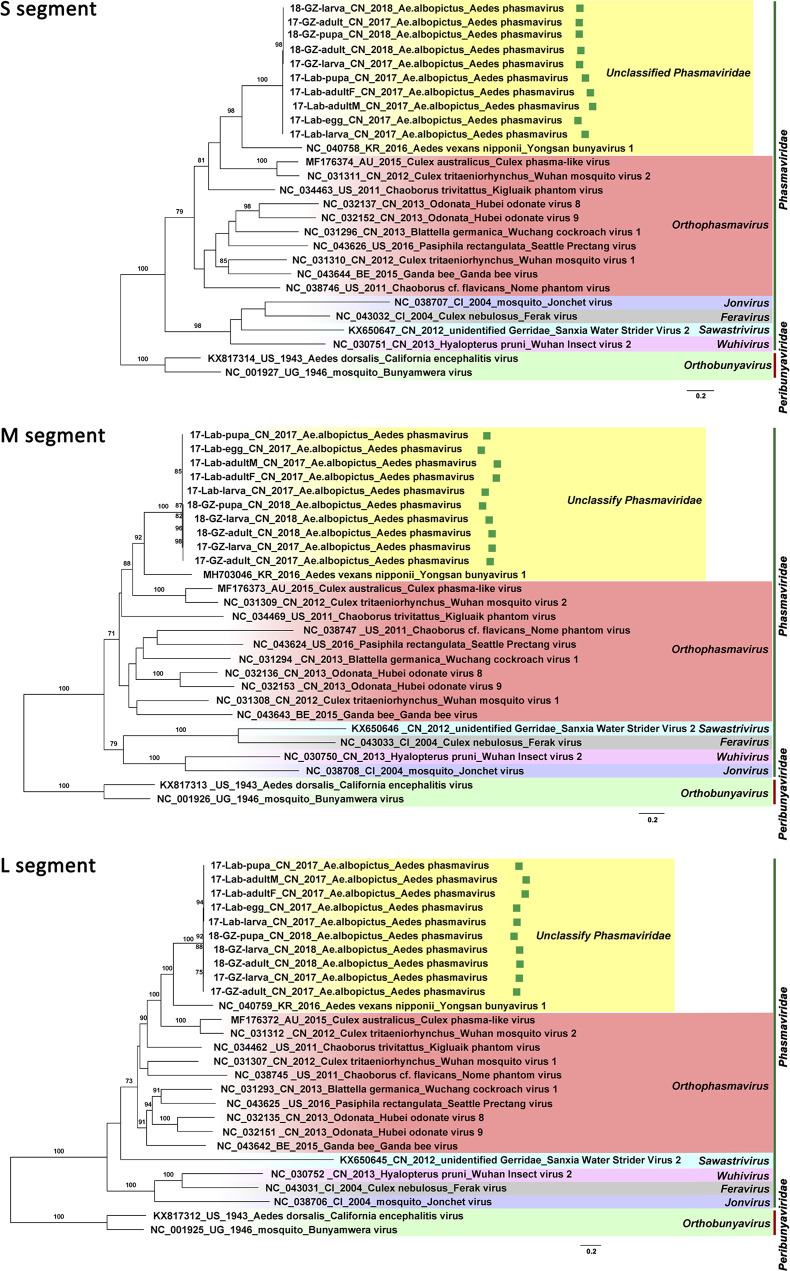
Maximum-likelihood phylogenetic tree based on the nucleotide level of complete coding region in the S, M, and L segments of the newly found *Aedes* phasmaviruses (APVs) in *Aedes* mosquitoes from China (highlighted with green squares) and other representative members in the family *Phasmaviriade.* Representatives in the genus *Orthobunyavirus* belonging to the family *Peribunyaviridae* were used as the outgroup.

In both lab- and field-derived *A. albopictus* sequencing data from samples collected in China in 2017 and 2018, three contigs of APV in each sample were found to share the highest amino acid identities of 50.8%, 48.1%, and 43.6% with the S, M, and L segments of YBV1 (GenBank accession no. MH703047.1, MH703046.1, and MH703045.1), respectively. In total, 10 viral genomes of APV with three segments were recovered from all sequenced *A. albopictus* samples (17-lab-egg, 17-lab-larva, 17-lab-pupa, 17-lab-adultM, 17-lab-adultF, 17-GZ-larva, 17-GZ-adult, 18-GZ-larva, 18-GZ-pupa, and 18-GZ-adult). The longest lengths were of 1,185, 2,022, and 6,468 nt, which corresponded to the nucleocapsid, glycoprotein, and RNA-dependent RNA polymerase (RdRp) genes, respectively. For all three segments, the nucleotide identity among the 10 assembled viral genomes ranged between 96% and 100%, which indicated that they represent closely related variants. In a separate phylogenetic analysis of the three segments, the 10 viral genomes of APV clustered together within the genus *Orthophasmavirus* in the family *Phasmaviridae* (Fig. 3). Their closest relative was YBV1, which was identified in *A. vexans* mosquitoes collected in South Korea in 2016, but with low levels of nucleotide similarity (ranging from 52% to 57%) in the coding regions of the three segments. Thus, the APVs present in the lab and field *A. albopictus* samples collected in China appeared to represent a novel species in the genus *Orthophasmavirus*.

### Phylogeny of Phasi Charoen-like phasivirus.

**FIG 4:**
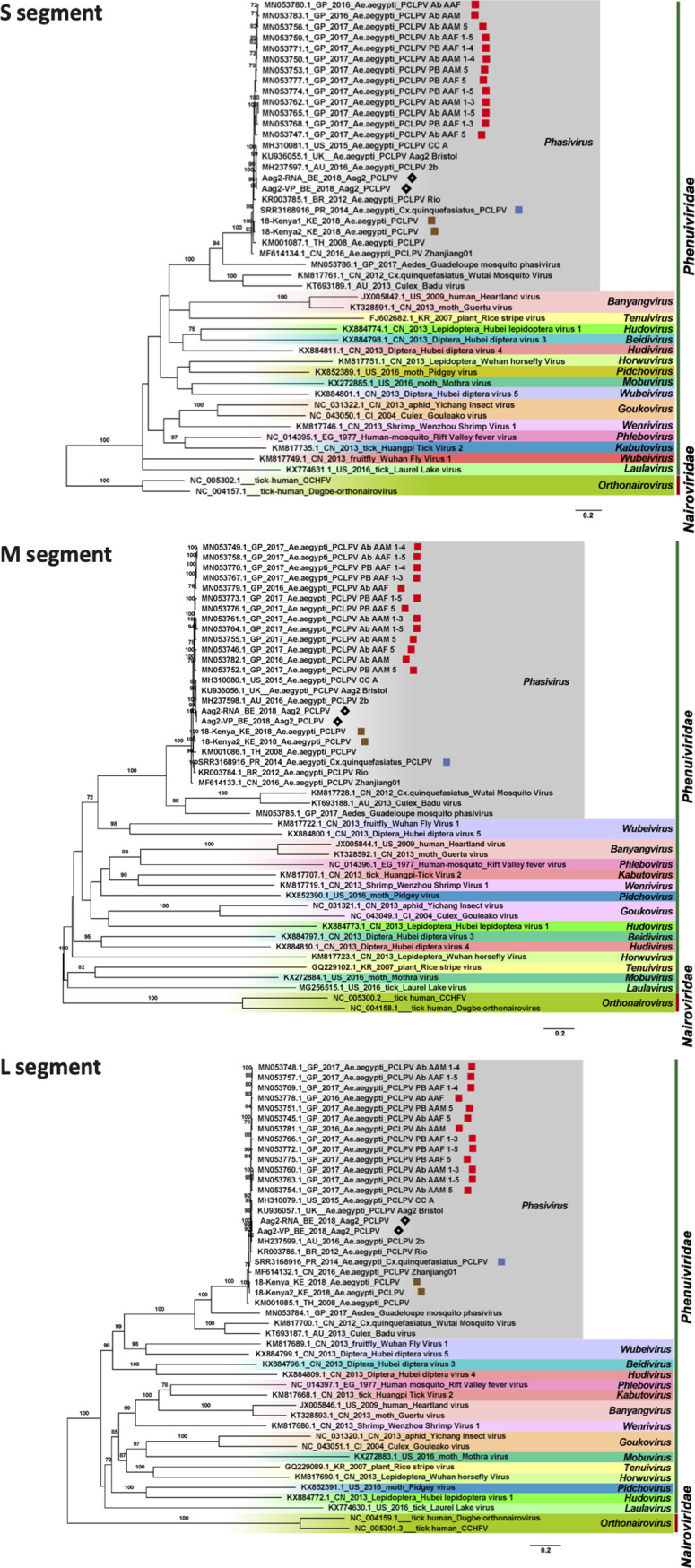
Maximum-likelihood phylogenetic tree based on the nucleotide sequence of complete coding regions of the S, M, and L segments of Phasi Charoen-like phasivirus (PCLPV) identified from *Aedes* mosquitoes and cells, together with other representative members of the family *Phenuiviriade.* The strains from Guadeloupe are highlighted with red squares, strains derived from Belgian Aag2 cells with black diamonds, strains from Kenya with brown squares, and strains from Puerto Rico with blue squares. Representative *Orthonairovirus* strains belonging to the family *Nairoviridae* were used as the outgroup.

Since the abundances of PCLPV reads in the *A. albopictus* samples from Guangzhou and lab-derived mosquitoes were relatively low, no complete genome was recovered from these pools. All three segments of PCLPV with a complete coding region were successfully recovered in one A. aegypti sample from Puerto Rico collected in 2014 and two A. aegypti samples from Kenya collected in 2018. The phylogenetic analysis was performed on these newly identified PCLPVs, on all other PCLPV genomes in GenBank (including those identified in A. aegypti mosquitoes collected in Thailand in 2008, in the United States in 2015, in Australia in 2016, in Zhejiang Province of China in 2016, in Guadeloupe in 2017, and in Aag2 cells from the United Kingdom), and on two more genomes obtained from lab Aag2 cell lines in Belgium (Fig. 4). It was interesting that all of the PCLPVs collected in distant geographic locations, in different years, and from field mosquitoes versus lab cell lines clustered very closely in the three maximum-likelihood (ML) trees of each segment. The nucleotide similarities among the genomes of PCLPVs were very high, ranging from 94% to 98% for the S segment, from 95% to 99% for M segment, and from 94% to 99% for L segment.

### Phylogeny of Guadeloupe mosquito virus-related viruses.

Twelve viral genomes (with complete coding regions) similar to GMV, Wenzhou sobemo-like virus 4, and Hubei mosquito virus 2 were obtained from the analyzed virome data sets, which included six pools of field *A. albopictus* collected in China (17-GZ-larvae, 17-GZ-adult, 18-GZ-larva, 18-GZ-pupa, 18-GZ-adult, and SRR7204303-CN-2016), three pools of an A. aegypti and *Cx. quinquefasciatus* mixture collected in Puerto Rico (SRR3168916-PR-2014, SRR3168921-PR-2014, and SRR3168924-PR-2014), and two A. aegypti pools collected in Kenya (SRR12102797-KE-2018 and SRR12102798-KE-2018) and one collected in Thailand (SRR6155879-TH-2015). The viral genomes contained two segments encoding an RdRp and one hypothetical protein on segment 1 (1,500 to 1,600 nt) and a capsid and one hypothetical protein encoded by segment 2 (3,000 to 3,200 nt). GMV, Wenzhou sobemo-like virus 4, and Hubei mosquito virus 2 are all newly described and currently unclassified viruses with a distant relationship to the families *Luteoviridae* and *Sobemoviridae* (28).

**FIG 5:**
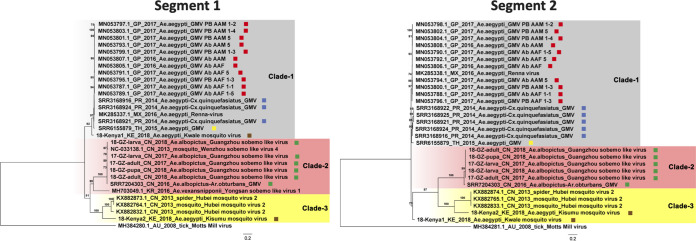
Maximum-likelihood phylogenetic tree based on the nucleotide sequence of the complete coding regions in segments 1 and 2 of Guadeloupe mosquito virus (GMV)-related viruses. The red, blue, yellow, brown, and green squares after the sequence names highlight the strains from Guadeloupe, Puerto Rico, Thailand, Kenya, and China, respectively. Motts Mill virus identified from ticks was used as the outgroup.

The phylogenetic analysis performed on the basis of segments 1 and 2 indicated that these sequences fell into three clades (Fig. 5). The genomes identified in samples collected in Thailand in 2015, Puerto Rico in 2014, and Kenya in 2018 clustered together with the Guadeloupe mosquito viruses found in Guadeloupe in 2016 and 2017, forming clade 1, with the nucleotide identity in this clade ranging from 90% to 99% for both segments. Five genomes recovered from *A. albopictus* collected in Guangzhou in 2017 and 2018, one recovered from the same host collected in Yunnan in 2016, and Wenzhou sobemo-like virus 4 clustered together in clade 2, with nucleotide identities among them ranging from 94% to 98%. The third clade consisted of Hubei mosquito virus 2 and one sequence from a sample collected in Kenya in 2018, with nucleotide identities ranging from 70% to 100%. The nucleotide similarities for the two segments among these three clades ranged from 54% to 69%.

## DISCUSSION

In this study, we first characterized the eukaryotic virome across different developmental stages of lab-derived and field-collected *A. albopictus* from China. Five pools from lab mosquitoes (containing 1,250 individuals in total) and five pools from field-collected mosquitoes (6,600 individuals in total) were analyzed (Table 1). Considering that the rearing conditions were stable in the lab and that the colony has been reared in the lab for many years, we collected lab-derived samples only in 2017 as no scientifically significant changes over time were anticipated. To take potential yearly virome fluctuations for field mosquitoes into account, they were collected in two consecutive years (2017 and 2018). The proportion of the eukaryotic viral reads in each sample (ranging from 0.2% to 15.8%) was comparable to that reported from previous mosquito virome studies (24, 28), suggesting that the sequencing depth was sufficient for virome analysis.

As expected, the virome diversity in lab *A. albopictus* was lower than that in field mosquitoes (Fig. 1), probably due to the less complex environment and the availability of clean water and food resources. Unlike the bacterial communities seen in field-collected mosquitoes (13, 31–35), it is interesting that the virome in lab-derived *A. albopictus* was very stable across all developmental stages, consistent with vertical transmission of these viruses. For the larvae only, relatively few viral reads were identified. This finding suggested that the virus might remain dormant at a very low concentration without or with very low rates of replication. The fact that these viruses were also present in field mosquitoes suggested that *A. albopictus* seems to contain a “vertically transmitted core virome” as was described before for A. aegypti and Culex quinquefasciatus from Guadeloupe (28), for which stable distinct core eukaryotic viromes were identified which possessed nearly identical viruses across time and space (28). In addition, another set of viruses was found to be shared by the field-collected *A. albopictus* mosquitoes across different stages, suggesting that they have a “core virome” with higher richness and diversity (Fig. S1). Whether these additional viruses were acquired from the environment or the lab-derived mosquitoes lost these viruses in captivity remains to be determined. All these vertically transmitted core viromes in *A. albopictus* deserve more attention with respect to their effects on vector competence for important medically relevant arboviruses. Due to the mosquito samples having originated from only one lab colony or one location in the field, further surveillance over a larger geographic range and longer periods of time will be needed to confirm the stability of the virome profile across different developmental stages. In addition, the larval pool collected in the wild in 2017 was an outlier, as it contained many unique viruses, which probably originated from the particular water environment they lived in, which could have been contaminated by other coinhabiting insects or larvae. Alternatively, it could be the case that a number of mosquitoes other than *A. albopictus* were included in the pool by accident. These results further suggest a significant influence of the breeding site ecology on the mosquito viral community.

Since some viruses (e.g., PCLPV, GMV, and Aedes aegypti totivirus) identified in *A. albopictus* from China were also identified as part of the core virome in A. aegypti from Guadeloupe (28), we were interested in determining how these core viruses in *Aedes* mosquitoes were further distributed around the world. Therefore, we explored 48 *Aedes* sp. viral metagenomic data sets from a public database (SRA). The samples were from different countries on different continents (China, the United States, Australia, Kenya, Thailand, Puerto Rico, and Guadeloupe) and from different mosquito species (A. aegypti, *A. albopictus*, *A. alboannulatus*, and *A. camptorhynchus*) and were treated with different wet-lab and sequencing procedures using different amounts of pooled mosquitoes and sequencing depths, but a highly prevalent set of widely distributed viruses, such as *Totiviridae*, *Orthomyxoviridae*, *Xinmoviridae*, *Flaviviridae*, PCLPV, and GMV, were able to be identified on the family or species level (Fig. 2). How these conserved viruses in *Aedes* mosquitoes interact with or influence an arbovirus infection is an interesting point for further studies. A previous study explored the effect of coinfections of PCLPV and cell-fusing agent virus (CFAV) on the replication of arboviruses in cell line Aa23 derived from *A. albopictus* (36). CFAV-PCLPV-positive Aa23 cells strongly inhibited the growth of two flaviviruses (ZIKV and DENV) and completely blocked infection by La Crosse virus (bunyavirus). Although the exact blocking mechanism was not known, on the one hand, the results suggested that the data generated from laboratory cell lines persistently infected by mosquito-specific viruses (MSVs) should be interpreted with caution. On the other hand, the intra-MSV interactions need to be considered when the influence of MSVs on vector competence is explored.

Although many viruses were very prevalent in *Aedes* mosquitoes, such as Chuvirus Mos8Chu0, Kaiowa virus, Wuhan mosquito virus 6, Whidbey virus, Aedes aegypti totivirus, and Australian Anopheles totivirus, phylogenetic analysis was further performed on GMV and PCLPV, which had the greatest number of complete coding genomes, and on APV, which was highly abundant in both field and lab *A. albopictus* from China. APV was found to be distantly related to the YBV1 identified from *A. vexans* from South Korea forming a separate clade. All the Chinese strains from both lab- and field-collected mosquitoes were very similar (Fig. 3). Also, for PCLPV, the genomes found in samples from different years, locations, and habitats were very similar for all three segments (Fig. 4). Interactions between bunyaviruses and arthropods occurring over 20 million years had been previously demonstrated (37–41). The findings indicating that APV and PCLPV seem to be very closely related were puzzling and might suggest a rather relatively recent spread of this virus or a very low evolutionary rate, which would be unexpected for RNA viruses. However, the effects of these viruses on their host are very poorly understood. A recent study revealed that preexisting infection of Aag2 cells with PCLPV did not affect the infection and growth of the mosquito-borne viruses in genus *Flavivirus*, *Alphavirus*, and *Rhabdovirus* in cell culture (42). GMV is a recently described two-segmented, currently unclassified virus. Three separate clades were observed for GMVs and GMV-related viruses, largely clustering according to location. This group of viruses should be proposed as a new family, and their role in arbovirus infection needs to be further studied.

In summary, our results reveal that the virome profile was very stable across different stages in both lab- and field-derived Aedes albopictus and that a number of possible core viruses exist in *Aedes* mosquitoes of at least four species in multiple locations in seven countries. Since the number of available *Aedes* virome data sets is still rather limited and since wet-lab procedures, sequencing depths, and pool sizes differed greatly among the analyzed data sets, the core viruses need to be further confirmed by next-generation sequencing (NGS) or PCR. To fully characterize and understand the genetic and phenotypic diversity of mosquito-specific viruses, samples from individual *Aedes* mosquitoes of additional species collected from additional locations and at additional time points, processed and sequenced with the same method, should be analyzed.

## MATERIALS AND METHODS

### Sample collection.

An *A. albopictus* colony was established in our laboratory (Wuhan, China) in 2017 which originated from a stable colony from the National Institute for Communicable Disease Control and Prevention (China CDC; Beijing, China). The adult mosquitoes were maintained at 27 to 30°C with 60% to 85% relative humidity using a photoperiod of a 12-h:12-h light-dark cycle. The larvae were fed with a mixture of yeast powder and wheat mill. Adult mosquitoes were placed into cages (30 cm by 30 cm by 30 cm), and the males were fed with 10% sucrose solution, while the females were fed with blood from mice. Egg, larva, pupa, and male and female adult samples of lab colony mosquitoes were collected in August 2017 (Table 1).

Field larva, pupa, and adult samples of *A. albopictus* were trapped in Guangzhou (Liwan district), Guangdong Province, China, in July to November of 2017 and 2018. In Kenya, adult mosquitoes of A. aegypti were trapped during July and August of 2018 in Ukunda and Kisumu. All samples were collected from residential quarters in urban areas, transported to the laboratory using an appropriate cold chain, and stored at −80°C. Mosquito species were determined by morphological identification, and samples were assigned into pools according to the date of collection (Table 1).

### Sample preparation for NGS.

The collected mosquitoes were pooled according to developmental stage for sequencing. The pooled samples were triturated by the use of a Tgrinder OSE-Y30 electric tissue grinder (Tiangen, China) on ice using sterile pestles with 200 μl of RPMI medium. Mosquito homogenates were clarified by centrifugation at 20,000 × *g* (4°C for 30 min) and filtered through a 0.45-μm-pore-size membrane filter (Millipore, Billerica, USA). Supernatants were collected and stored at −80°C. RNA was extracted from the supernatants with an RNeasy minikit (Qiagen, Germany) according to the manufacturer’s instructions. Then, strand-specific libraries were prepared using a TruSeq stranded total RNA sample preparation kit (Illumina, USA). TruSeq PE (paired-end) cluster kit v3 (Illumina, USA) and TruSeq SBS kit v3-HS (Illumina, USA) (300 cycles) were used for sequencing with 150 bp per read (PE 2 × 150 bp), which was performed on an Illumina HiSeq 2500 platform (Illumina, USA) by Shanghai Biotechnology Corporation.

### Bioinformatic analysis of viral metagenomic data.

The obtained raw paired-end reads were trimmed for quality and adapters using Trimmomatic (43), and the remaining reads were *de novo* assembled into contigs using metaSPAdes (44). Contigs from all pools longer than 500 bp were filtered for redundancy at 95% nucleotide identity over 80% of the length using ClusterGenomes (45). The collection of representative contigs was classified using DIAMOND (46) against the nr database (updated 29 September 2019) using the sensitive mode for taxonomic annotation. KronaTools (47) was used to parse the output file of DIAMOND, which was used to find the least common ancestor of the best 25 DIAMOND hits (based on BLASTx score) for each contig. All contigs annotated as eukaryotic viruses were extracted using an in-house Python script. The abundance and length coverage of eukaryotic virus contigs in individual pools were obtained by aligning trimmed and decontaminated reads of each sample to the collection of representative contigs using BBMap (48). Abundance tables were extracted from eukaryotic viruses and further used for making heat maps in R with the ComplexHeatmap (49), ggplot2 (50), and phyloseq (51) packages. The length coverage of each viral species per sample was calculated by the following formula: contig length covered by at least one read/the total length of the corresponding contig.

### Eukaryotic virus screening of *Aedes* mosquito virome data in SRA.

**FIG 6:**
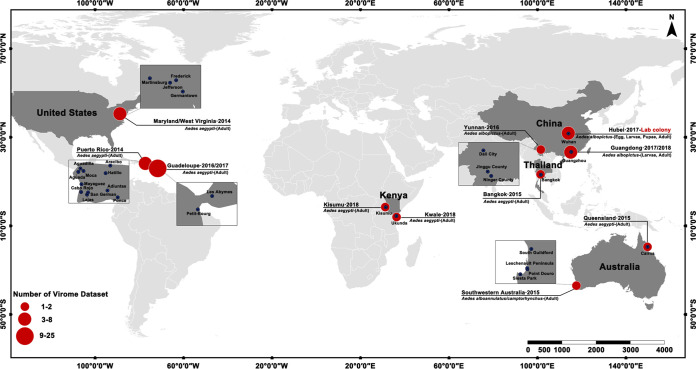
Global distribution of *Aedes* mosquitoes virome data sets used in this study.

To investigate the level of conservation of the eukaryotic core viruses identified in Chinese samples from this study with those of other *Aedes* mosquitoes worldwide, we retrieved 48 published SRA data sets (including 25 data sets of *Aedes* sp. from Guadeloupe 28], 8 from Puerto Rico [16], 4 from the United States [16], 7 from Australia [52, 53], 2 from Kenya, 1 from Thailand [53], and 1 from China [54]) (Fig. 6; see also Table S1 in the supplemental material). The world map displaying the geographic origin of all *Aedes* virome data sets used in this study was drawn with ArcGI (ArcMap 10.5). The raw reads of the SRA data sets were *de novo* assembled using SKESA (55) with default settings, which was less computationally intensive than metaSPAdes. These obtained contigs were taxonomically annotated using DIAMOND against the nr database (updated 29 September 2019) in sensitive mode. KronaTools (47) and an in-house Python script were used to parse the output file of DIAMOND and extract eukaryotic virus contigs.

### The presence of eukaryotic viruses in all *Aedes* virome data sets.

Taxonomically identified eukaryotic virus contigs longer than 500 bp were extracted from all 58 *Aedes* virome data sets and placed together in a single file. Viral species that contained at least one contig longer than 1,500 bp were used for visualization purposes. A viral species was considered present in the sample as long as the sample had one contig (>500 bp) assigned to the species. The viral species shared among different countries were visualized with an UpSet plot (Fig. 2A) using the UpSetR package (56). The viral species are shown in the heat map (Fig. 2B) only if they were present in samples from at least three countries.

### Phylogenetic analysis.

To obtain the complete genomes related to Yongsan bunyavirus 1 (YBV1), Phasi Charoen-like phasivirus (PCLPV), and Guadeloupe mosquito virus (GMV) from all *Aedes* virome data sets, the trimmed reads of each sample were individually mapped with BWA (57) against the following selected reference genomes: (i) 17-Lab-egg-L (MT361040), 17-Lab-egg-M (MT361040), and 17-Lab-pupa-S (MT361044) as the reference for YBV1; (ii) PCLPV Rio strain (KR003786.1, KR003784.1, and KR003785.1) for PCLPV; and (iii) 18-GZ-larva-seg1 (MT361057) and 18-GZ-pupa-seg2 (MT361060) for GMV. The consensus sequences of these viruses were generated from the bam files using SAMtools and bcftools (58).

The nucleotide sequences of the complete genomes or complete coding regions in the genomes (segments) of these viruses were used to determine their evolutionary history together with appropriate reference strains from GenBank. Alignments of the sequences were performed with MAFFT v7.222 (59) using the most accurate algorithm, L-INS-I, with 1,000 cycles of iterative refinement. Ambiguously aligned regions were removed by trimAl v1.2 (60) using an automated trimming heuristic, which was optimized for maximum-likelihood (ML) phylogenetic tree reconstruction. The phylogenetic trees for each segment were reconstructed from 1,000 ultrafast bootstrap ML tree replicates using IQ-TREE v1.6.11 (61) with best-fit model selection by ModelFinder (62). FigTree v1.4.3 (63) was used for phylogenetic tree visualization.

### Data availability.

Accession numbers of the viruses obtained from our data set are listed in Table S2, and viral genome sequences recovered from the SRA data sets can be found at https://github.com/Matthijnssenslab/AedesVirome. All viral sequences used to construct phylogenetic trees can be found at https://github.com/Matthijnssenslab/AedesVirome.

10.1128/mSystems.00640-20.3 TABLE S2 Accession numbers of YBV1-, PCLPV-, and GMV-related viruses identified in this study. Download Table S2, XLSX file, 0.01 MB. Copyright © 2020 Shi et al. 2020 Shi et al. This content is distributed under the terms of the Creative Commons Attribution 4.0 International license.
