# Supplementary figures and images for: Stability of the Virome in Lab- and Field-Collected Aedes albopictus Mosquitoes across Different Developmental Stages and Possible Core Viruses in the Publicly Available Virome Data of Aedes Mosquitoes
Source: mSystems. 2020 Sep 29;5(5):e00640-20. doi: 10.1128/mSystems.00640-20 (PMC7527137; doi:10.1128/mSystems.00640-20)

Alpha Diversity Measure

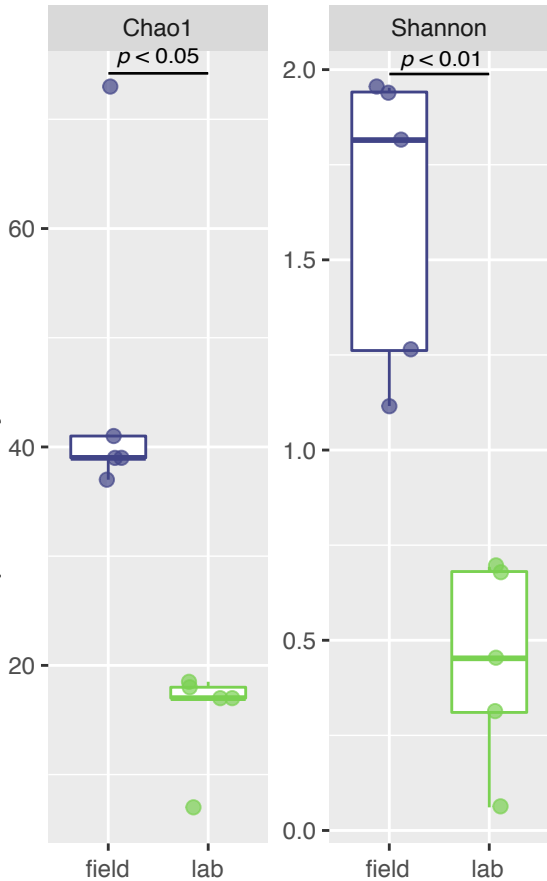

Supplement: FIG S1 [file mSystems.00640-20-sf001.pdf]
